# Taxonomy, Ontogenesis and Evolutionary Relationships of the Algae-Bearing Ciliate *Bourlandella viridis* (Kahl, 1932) comb. nov., With Establishment of a New Genus and New Family (Protista, Ciliophora, Hypotrichia)

**DOI:** 10.3389/fmicb.2020.560915

**Published:** 2021-01-28

**Authors:** Wenya Song, Tengyue Zhang, Xue Zhang, Alan Warren, Weibo Song, Yan Zhao, Xiaotian Luo

**Affiliations:** ^1^Institute of Evolution and Marine Biodiversity, and College of Fisheries, Ocean University of China, Qingdao, China; ^2^Key Laboratory of Aquatic Biodiversity and Conservation of Chinese Academy of Sciences, Institute of Hydrobiology, Chinese Academy of Sciences, Wuhan, China; ^3^Department of Zoology, Comenius University in Bratislava, Bratislava, Slovakia; ^4^Department of Life Sciences, Natural History Museum, London, United Kingdom; ^5^College of Life Sciences, Capital Normal University, Beijing, China

**Keywords:** algal endosymbiosis, *Bourlandella viridis* gen. nov., comb. nov., Bourlandellidae fam. nov., dorsomarginalia, systematics

## Abstract

Hypotrichs are the most complex and highly differentiated ciliate lineages and have great potential for evolutionary novelties. Problems in hypotrich systematics are mainly due to discordance between the morphological and genetic data (mainly small subunit rRNA gene). Species with morphologies that are characteristic of two or more higher rank taxa are probably a major contributing factor to these conflicts. The present study describes a Chinese population of a poorly known organism with numerous endosymbiotic zoochlorellae, the morphology of which corresponds well with the type population of *Limnoholosticha viridis* (Kahl, 1932) Li et al., 2017. Newly obtained information shows this species has a zigzag midventral cirral pattern that is diagnostic of urostylids, whereas the dorsal ciliature shares features (presence of dorsomarginal kinety and dorsal kinety 3 fragmentation) that are typical of oxytrichids. Molecular phylogenetic analyses reveal a close relationship with oxytrichids. An integrative approach combining morphological, morphogenetic, phylogenetic and ecological analyses indicates that *L. viridis* represents a new genus and new family which might be an intermediate form between uorstylids and dorsomarginalians. Thus, Bourlandellidae fam. nov. and *Bourlandella* gen. nov. are here established. Lastly, we speculate that phenotypic convergence and mixtrophy might confer on the new combination, *Bourlandella viridis* (Kahl, 1932) comb. nov., the ability to adapt to a wide range of environmental conditions.

## Introduction

Ciliated protists (ciliates) are a large group of unicellular eukaryotes, many of which have ubiquitous distributions (Kahl, 1932; Corliss, 1979; Lynn, 2008; Song et al., 2009; Hu et al., 2019), that have long been used as model organisms in studies of cytology, evolutionary biology and ecology (Foissner et al., 2008; Clark and Peck, 2009; Gao et al., 2016; Stoeck et al., 2018; Chen et al., 2019; He et al., 2019; Wang et al., 2019). Hypotrichs (subclass Hypotrichia s. str.) are widely regarded as the most complex and highly differentiated ciliate group (Fauré-Fremiet, 1961; Borror, 1972; Jankowski, 1979; Foissner, 1982; Tuffrau and Fleury, 1994; Berger, 1999, 2006, 2008, 2011; Jung and Berger, 2019; Shao et al., 2019). Nevertheless, the hypotrichs remain one of the most confused groups in terms of their systematics, mainly due to a lack of morphological, ontogenetic and molecular data for many taxa (Kaur et al., 2019; Kim and Min, 2019; Park et al., 2020; Zhang et al., 2020). Two well established groups among the hypotrichs are the order Urostylida Jankowski, 1979 and family Oxytrichidae Ehrenberg, 1830. Urostylids are characterized by their midventral cirri arranged in a zigzag pattern (Berger, 2006), while oxytrichids are characterized by having 18 frontal-ventral-transverse (FVT) cirri and dorsomarginal kineties with dorsal kinety fragmentation, although this latter feature is absent in *Uroelptus* Ehrenberg, 1831, *Rigidothrix* Foissner and Stoeck, 2006 and *Rigidosticha* Bharti et al., 2016 (Berger, 1999). However, morphological and genetic discordances have frequently been reported, especially in species that exhibit intermediate morphological characteristics such as the possession both of midventral cirri arranged in a zigzag pattern and dorsomarginal kineties with or without dorsal kinety fragmentation (Foissner and Stoeck, 2006, 2008; Foissner et al., 2010; He et al., 2011; Bharti et al., 2016; Chen et al., 2016). The CEUU (Convergent Evolution of Urostylids and Uroleptids) hypothesis was proposed by Foissner et al. (2004) to reconcile traditional classifications with molecular data. According to this hypothesis, the morphological similarity and the genetic divergence between uroleptids and urostylids are due to convergent evolution of the zigzag midventral pattern, which might have evolved twice from the 18 FVT cirral pattern by anlagen multiplication.

*Holosticha* Wrześniowski, 1877, the name-bearing type genus of the Holostichidae Fauré-Fremiet, 1961, has traditionally been a melting pot for species with three distinct frontal cirri, a midventral complex composed of cirral pairs only, transverse cirri and one marginal row on each side of the body (Wrześniowski, 1877; Berger, 2003, 2006). Berger (2003) split *Holosticha* into four genera and transferred the species with caudal cirri into *Caudiholosticha* Berger, 2003. More recently, newly obtained molecular and ontogenetic data indicated that the type species of *Caudiholosticha*, *C*. *stueberi* (Foissner, 1987) Berger, 2003, should be assigned to *Uroleptus* Ehrenberg, 1831 and another 16 species in the genus were tentatively classified into six new genera including *Caudiholosticha viridis* (Kahl, 1932) Berger, 2003 which was assigned to the genus *Limnoholosticha* Li et al., 2017 (Li et al., 2017). The genus *Limnoholosticha* was established for urostylid species discovered from limnetic habitats and having the following morphological characteristics: elliptical to wide-elliptical outline; three frontal cirri, buccal cirrus and parabuccal cirrus present, about five to eight transverse cirri; midventral complex likely composed of pairs only; one left and one right marginal row; caudal cirri present, presence/absence of frontoterminal and pretransverse ventral cirri not known. *Limnoholosticha viridis* was first reported by Kahl (1932) as *Holosticha viridis.* It was next reported by Shen (1983) who gave an incomplete description based on *in vivo* observations. However, due to a lack of information on its ciliature pattern, morphogenesis and molecular phylogeny, the taxonomy and systematics of *L. viridis* are uncertain.

During a survey of the ciliate biodiversity of northeastern China, we isolated a population which matches *L. viridis*, giving us the opportunity to investigate its morphology, morphogenesis, ecology and molecular phylogeny and to revise its taxonomy. Based on these findings, we also discuss the systematics of *Holosticha*-like species and re-examine their evolutionary relationships. Finally, we explore the origins of the zigzag pattern of ventral cirri among hypotrichs from a phylogenetic perspective in order to gain a better understanding of this morphological feature in taxa with high genetic variations.

## Materials and Methods

### Sample Collection, Isolation, and Identification

*Bourlandella viridis* (Kahl, 1932) comb. nov. was isolated on February 26, 2019 from a freshwater pond in Baihua Garden, Qingdao, China (36°04′11″N, 120°21′05″E), when the water temperature was 4°C. Raw cultures were established at room temperature (about 25°C) using Petri dishes filled with habitat water. A clonal culture was subsequently established from a clone-founding cell isolated from a raw culture. Several squashed wheat grains were added to support the growth of bacteria which served as a food source for the ciliates in clonal and raw cultures. About ten cells were selected from the clonal culture, washed four times, and transferred into Petri dishes with Volvic mineral water and without additional bacterial food, for a program of phylogenomic analyses. After two weeks, cells were isolated for DNA extraction.

Cells were observed *in vivo* using bright field and differential interference contrast microscopy, and their infraciliature was revealed using the protargol staining method according to Wilbert (1975). *In vivo* measurements were conducted at magnifications of 100× to 1,000×. Drawings of live cells were based on free-hand sketches, and those of protargol-stained specimens were made at 1,000× magnification with the help of a drawing device. To illustrate the changes that occur during morphogenetic processes, old (parental) ciliary structures are depicted by contour while new structures are shaded black. Terminology and systematics mainly follow Berger (1999, 2006).

### DNA Extraction, PCR Amplification, and Sequencing

Single cells were isolated from the clonal culture and washed four times using autoclaved ultrapure water to remove potential contaminants. DNA extraction was performed following procedures described in Huang et al. (2018). The amplification of the SSU rRNA gene was conducted according to Sheng et al. (2018) using the primers 18S-F (5′-AACCTGGTTGATCCTGCCAGT-3′) and 18S-R (5′-TGATCCTTCTGCAGGTTCACCTAC-3′) (Medlin et al., 1988). To minimize the possibility of amplification errors, Q5 Hot Start High-Fidelity DNA Polymerase (New England BioLabs, United States) was used. Sequencing was performed bidirectionally in five reactions by the Tsingke Biological Technology Company (Qingdao, China).

### Molecular Data and Phylogenetic Analyses

The SSU rRNA gene sequence derived from *B. viridis* was assembled by using Seqman (DNAStar) and formed the dataset with another 105 related gene sequences downloaded from the GenBank database (see Figure 6 for accession numbers). Four strombidiids, namely *Novistrombidium orientale* (FJ422988), *Strombidium apolatum* (DQ662848), *Strombidinopsis acuminata* (FJ790209), and *Parastrombidinopsis minima* (DQ393786), were selected as the outgroup taxa. All the sequences were aligned using the GUIDANCE web server^1^ (Penn et al., 2010). Both ends of the alignment were trimmed manually using Bioedit v.7.2.6 (Hall, 1999). The final alignment included 106 taxa and 1,801 sites among which 591 were variable sites and 390 were parsimony-information sites.

The SSU rRNA gene-based phylogenetic position of *B. viridis* was investigated by maximum likelihood (ML) and Bayesian inference (BI) analyses. The GTR evolutionary model was selected based on the Bayesian Information Criterion (BIC) and Akaike Information Criterion (AIC) in jModelTest v.2.1 (Darriba et al., 2012). The ML tree was generated using RAxML-HPC2 v.8.2.12 on XSEDE with 1,000 bootstrap replicates under the GTRGAMMA model in the CIPRES Science Gateway^2^. BI analysis was carried out with MrBayes v.3.2.6 on XSEDE using the best model GTR+I+G via the online CIPRES Science Gateway web server (Miller et al., 2010; Ronquist et al., 2012; Stamatakis, 2014). Posterior distributions of trees were sampled using Markov chain Monte Carlo (MCMC) simulations running for 10,000,000 generations with a sampling frequency of 100. The first 25% of trees were discarded as burn-in. Trees were visualized in MEGA v.7.0 (Kumar et al., 2016).

## Results

### ZooBank Registration

Present work: LSIDurn:lsid:zoobank.org:pub:B2CF810F-9B8F-4BE5-9F1D-B2E2AB7C0941.

Bourlandellidae fam. nov.: LSIDurn:lsid:zoobank.org:act: FE6717A6-4D5D-4B78-A478-84B929EC4574.

*Bourlandella* gen. nov.: LSIDurn:lsid:zoobank.org:act:3DCE8 CA5-32E7-4116-9DE1-3B77BD0A9254.

*Bourlandella viridis* (Kahl, 1932) comb. nov.: LSIDurn:lsid: zoobank.org:act:FF84A05E-9A66-4DC9-B19E-F11261D4A0D4.

Subclass Hypotrichia Stein, 1859

Dorsomarginalia Berger, 2006

Family Bourlandellidae fam. nov.

#### Diagnosis

Dorsomarginalia with midventral complex composed of cirral pairs only; three frontal cirri; frontoterminal cirri present; dorsal kinety 3 with simple fragmentation; dorsomarginal rows present; ontogenesis in urostylid-mode with FVT-cirri formed in more than six anlagen.

#### Etymology

The new family name is derived from the type genus *Bourlandella*.

#### Type Genus

*Bourlandella* gen. nov.

#### Genera Assignable

*Bourlandella* gen. nov.

Genus *Bourlandella* gen. nov

#### Diagnosis

Bourlandellidae with midventral complex extending to posterior half of cell; buccal cirrus, pretransverse ventral and transverse cirri present; one left and one right marginal row; caudal cirri present; dorsal kinety fragmentation in *Oxytricha* pattern; two well-developed dorsomarginal kineties; old adoral zone of membranelles completely retained by the proter during cell division.

#### Dedication

We dedicate this new genus to our eminent colleague, Dr. William A. Bourland, Boise State University, USA, in recognition of his contributions to ciliatology.

#### Type Species

*Bourlandella viridis* (Kahl, 1932) comb. nov.

#### Species Assignable

*Bourlandella viridis* (Kahl, 1932) comb. nov.

*Bourlandella viridis* (Kahl, 1932) comb. nov.

#### Synonyms

*Holosticha viridis* Kahl, 1932

*Caudiholosticha viridis* (Kahl, 1932) Berger, 2003

*Limnoholosticha viridis* (Kahl, 1932) Li et al., 2017

#### Remarks

*Bourlandella viridis* was first reported by Kahl (1932) as *Holosticha viridis.* Shen (1983) gave an incomplete redescription based on a Tibetan population. An improved diagnosis is supplied here based on previously described populations and the Qingdao population. A redescription is supplied based on the Qingdao population.

#### Improved Diagnosis of *Bourlandella viridis* (Kahl, 1932) comb. nov.

Body 100–130 × 50–60 μm *in vivo*, ovoidal to broad elliptical in shape; cells often yellow-greenish or greenish due to the presence of algal inclusions; cortical granules absent; adoral zone occupies about one-third of body length, with about 27 membranelles; three frontal, one buccal, two frontoterminal, two or three pretransverse ventral, and on average nine transverse cirri; midventral complex with on average eight midventral pairs, extends to anterior end of transverse cirral row; posterior end of marginal rows confluent; six dorsal kineties (DK) including two dorsomarginal rows; two macronuclear nodules, each closely associated with a micronucleus; endosymbiotic algae present; freshwater habitat.

#### Voucher Slides

Fourteen permanent voucher slides with multiple protargol-stained individuals are deposited in the Laboratory of Protozoology, Ocean University of China, Qingdao, China (registration no. SWY2019022601/1-14).

### Morphological Redescription

**FIGURE 1:**
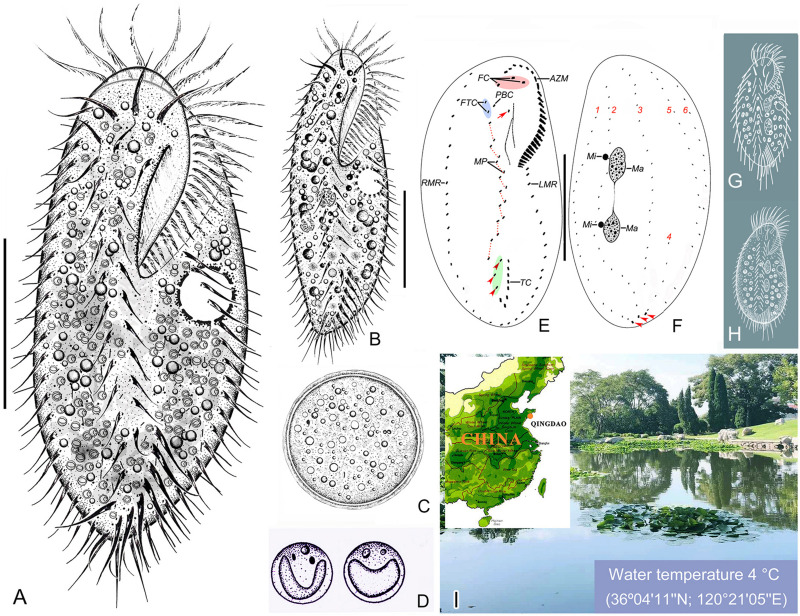
Morphology and infraciliature of *Bourlandella viridis* (Kahl, 1932) comb. nov. *in vivo* **(A–D,G,H)** and after protargol staining **(E,F)**. **(A)** Ventral view of a representative individual from raw culture. **(B)** Ventral view of an individual from a culture that was maintained in the laboratory for a prolonged period of time. **(C)** Resting cyst. **(D)** Endosymbiotic green algae. **(E,F)** Ventral **(E)** and dorsal **(F)** views of the same individual, showing infraciliature and nuclear apparatus, arrow marks buccal cirrus, arrowheads in **(E)** denote pretransverse ventral cirri, arrowheads in **(F)** mark caudal cirri. **(G)** *B. viridis* (after Kahl, 1932, called *Holosticha viridis*). **(H)** *B. viridis* (after Shen, 1983, called *Holosticha viridis*). **(I)** Location of the sample site. AZM, adoral zone of membranelles; FC, frontal cirri; FTC, frontoterminal cirri; LMR, left marginal row; Ma, macronuclear nodules; Mi, micronuclei; MP, midventral pairs; PBC, parabuccal cirrus; RMR, right marginal row; TC, transverse cirri; 1–6, dorsal kineties 1–6. Bars: 40 μm.

**FIGURE 2:**
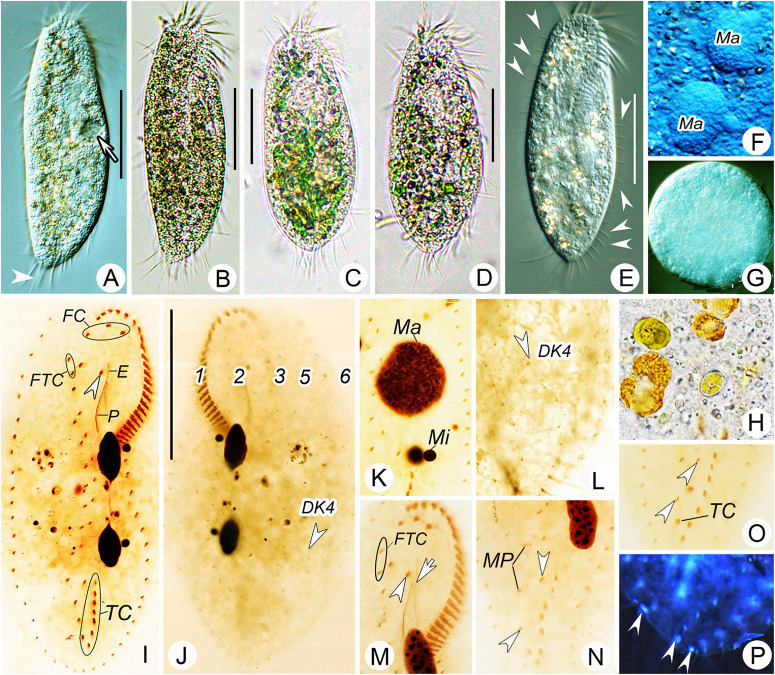
Morphology of *Bourlandella viridis* (Kahl, 1932) comb. nov. from life (**B–D**, bright field; **A,E–G**, differential interference contrast) and after protargol staining **(I–P)**. **(A,B,E)** Ventral views of different individuals from clonal culture, arrow in **(A)** indicates contractile vacuole, arrowhead in **(A)** marks the long caudal cirrus, arrowhead in **(E)** shows the elongated dorsal bristles. **(C,D)** Ventral view of different individuals from raw culture. **(F)** Details of macronuclear nodules in ventral view. **(G)** Resting cyst. **(H)** Food vacuole containing algae. **(I,J)** Ventral **(I)** and dorsal **(J)** views of the same specimen, showing infraciliature and nuclear apparatus, arrowhead in **(I)** indicates buccal cirrus, arrowhead in **(J)** marks dorsal kinety 4 (DK4). **(K)** Details of nuclear apparatus, to show numerous nucleoli. **(L)** Dorsal view, showing DK4 fragmented from DK3. **(M–O)** Ventral views, showing infraciliature, arrowhead and arrow in **(M)** marks buccal cirrus and undulating membrane, respectively, arrowheads in **(N)** and **(O)** show two pretransverse ventral cirri. **(P)** Ventral view with inverting color by Photoshop CS5, showing the caudal cirri (arrowheads). DK4, dorsal kinety 4; E, endoral; FC, frontal cirri; FTC, frontoterminal cirri; Ma, macronuclear nodules; Mi, micronuclei; MP, midventral pairs; P, paroral; TC, transverse cirri; 1–3, 5, and 6, dorsal kineties. Bars: 40 μm.

Cells from raw cultures 100–130 × 50–60 μm *in vivo*, body length:width ratio 2.0–2.5:1 (*n* = 4), ovoidal to broad elliptical with slightly tapered posterior end (Figures 1A, 2C,D); cells from clonal cultures slightly more slender (Figures 1B, 2A,B,E), body 90–120 × 30–40 μm *in vivo*, length to width ratio 2.5–3.0:1 (*n* = 4). Body flexible but non-contractile. Cytoplasm colorless, containing numerous green spherical endosymbiotic algae (1.5–2.5 μm in size; distributed evenly throughout the cell apart from at location of macronuclear nodules; algae absent from cells kept in laboratory culture for prolonged periods of time, i.e., about 1 month), food vacuoles filled with algae (in cells from raw cultures and disappeared after several days in clonal culture), and small irregular crystals, giving cells yellow-greenish or greenish appearance (Figures 1A,D, 2C,D). Contractile vacuole approximately 12 μm across at the end of diastole, located at or slightly anterior of equatorial region, near left body margin (Figures 1A,B, 2A). Nuclear apparatus located at about middle third of body length, comprising two spherical to ellipsoidal macronuclear nodules, each with numerous small nucleoli; in protargol preparations, anterior nodule about 21 × 12 μm, posterior nodule about 23 × 12 μm; each nodule with a closely associated, relatively large (2–6 μm) micronucleus (Figures 1F, 2F,I–K). Locomotion by continuous rapid crawling on bottom of Petri dish and on surface of water.

Resting cysts formed after several days in culture. Cysts spherical in shape, about 50 μm in diameter. Cyst wall comprising a compact external layer about 0.8 μm thick and an inner layer about 1.5 μm thick. Cyst cytoplasm usually filled with colorless granules about 1.5–2.5 μm across (Figures 1C, 2G). Whether the macronuclear nodules in the cysts are fused could not be determined.

Adoral zone extends to about 33% of body length *in vivo* and about 36% of body length in protargol-stained cells, composed of on average 27 membranelles with cilia up to 16 μm long. Paroral slightly longer than endoral, anterior end commences at about 30% length of endoral and may or may not optically intersect with endoral (paroral and endoral intersected in five out of 25 cells examined) (Figures 1D, 2I,M). Three enlarged frontal cirri behind distal portion of adoral zone (Figures 1E, 2I). Buccal cirrus located ahead of paroral, to right of endoral and slightly behind level of parabuccal cirrus (III/2) (Figures 1E, 2I,M). Constantly two frontoterminal cirri located to right of parabuccal cirrus (Figures 1E, 2I,M). Midventral complex composed of about eight pairs of cirri arranged in a typical zigzag pattern, terminates near anterior end of transverse cirral row (Figures 1E, 2N). Usually two pretransverse ventral cirri (three pretransverse ventral cirri present in five out of 30 cells examined) (Figures 1E, 2I,N,O). Seven to ten transverse cirri in J-shaped pseudorow; rearmost cirri protrude beyond posterior body margin (Figures 1A,B,E, 2I,O). Single marginal row on each side of body, posterior ends confluent; right row commences near distal end of adoral zone, left row commences at level of cytostome (Figures 1A,B,E, 2I and Table 1).

**TABLE 1:** Morphometric characterization of *Bourlandella viridis* (Kahl, 1932) comb. nov. based on the Qingdao population.

Character	Min	Max	Mean	M	SD	CV	*n*
Body, length	90	156	119.0	121	16.8	14.1	25
Body, width	35	88	55.2	49	15.1	27.7	25
AZM, length	24	57	42.6	42	7.8	18.4	25
AZM, width	19	36	26.6	25	4.7	17.8	25
Length of AZM: body length, ratio (%)	26	47	37	38	5.20	14.16	25
Paroral, length	15	22	21.5	21	3.2	14.9	23
Endoral, length	14	29	20.9	20	3.7	17.8	25
Length adoral zone: length paroral, ratio (%)	32	74	50.0	50	10.5	21.1	23
Anterior end to buccal cirrus, distance	19	36	24.7	24	3.7	15.1	25
Anterior body end to first frontoterminal cirrus, distance	18	25	24.7	25	3.9	15.8	25
Anterior body end to second frontoterminal cirrus, distance	23	41	30.0	29	4.4	14.8	25
Anterior body end to right marginal row, distance	8	22	13.7	13	3.4	25.1	25
Posterior body end to posteriormost transverse cirrus, distance	3	17	10.0	9.0	3.1	30.9	24
Anterior body end to first macronuclear nodule, distance	28	57	36.7	36	7.0	19.0	25
Adoral membranelles, number	22	35	26.9	27.0	3.2	11.2	32
Frontal cirri, number	3	3	3.0	3	0.0	12.0	25
Buccal cirrus, number	1	1	1.0	1	0.0	0.0	25
Parabuccal cirrus, number	1	1	1.0	1	0.0	0.0	25
Frontoterminal cirri, number	2	2	2.0	2	0.0	0.0	25
Midventral pairs, number	6	11	8.1	8	1.1	13.8	25
Transverse cirri, number	7	10	8.6	9	1.0	11.9	22
Pretransverse ventral cirri, number	2	3	2.2	2.0	0.4	17.2	30
Left marginal cirri, number	15	25	21.0	21.0	2.9	14.1	20
Right marginal cirri, number	19	30	24.4	23.5	3.9	16.1	20
Caudal cirri, number	3	3	3.0	3	0.0	0.0	25
Dorsal kineties, number	6	6	6.0	6.0	0.0	0.0	20
Dikinetids in dorsal kinety 1, number	18	29	22.7	21.0	2.9	12.9	16
Dikinetids in dorsal kinety 2, number	18	24	21.2	21.0	2.0	9.6	16
Dikinetids in dorsal kinety 3, number	14	21	18.5	19.0	2.2	11.8	16
Dikinetids in dorsal kinety 4, number	5	8	6.5	6.0	1.1	16.6	16
Dikinetids in dorsal kinety 5, number	11	20	16.4	16.0	2.2	13.5	16
Dikinetids in dorsal kinety 6, number	7	13	9.5	9.0	1.7	18.1	16
Total number of dorsal dikinetids	85	101	85	93.9	94	5.8	16
Macronuclear nodules, number	2	2	2.0	2	0.0	0.0	21
Anterior macronuclear nodule, length	11	29	21.7	24	6.1	26.5	21
Anterior macronuclear nodule, width	6	20	12.1	12	3.6	30.0	21
Posterior macronuclear nodule, length	13	37	23.6	25	6.3	26.8	21
Posterior macronuclear nodule, width	5	21	12.2	12	4.7	38.9	21
Micronuclei, number	2	2	2.0	2.0	0.0	0.0	18
Micronucleus, length	2	6	4.4	5.0	1.2	27.2	18
Micronucleus, width	2	6	3.7	4.0	1.3	36.2	18

*All data based on protargol-stained specimens. Measurements in μm. AZM, adoral zone of membranelles. CV, coefficient of variation in %. M, median. Max, maximum. Mean, arithmetic mean. Min, minimum. n, number of cells measured. SD, standard deviation.*

Dorsal ciliature in *Oxytricha* pattern, invariably with six DK (Figures 1F, 2J). DK1 (leftmost dorsal kinety) shortened anteriorly and reaches posterior end of body. DK2 extends almost entire body length. DK3 commences at anterior end of body and terminates posteriorly about 85% body length. DK4 commences about 65% down body length and terminates at posterior end of body. Two dorsomarginal kineties (DK5 and DK6); DK5 composed of 11–20 (on average 16) bristles, extends nearly to posterior end of body; DK6 composed of 7–13 (on average 9) bristles, terminates posteriorly slightly below equator of cell. Dorsal bristles conspicuous when viewed using differential interference contrast microscopy, about 3–7 μm long, posterior bristles much longer than anterior bristles (Figures 1F, 2J,L). Three long caudal cirri located at posterior body margin, one each at posterior end of DK1, 2 and 4; cilia about 14–16 μm long; readily distinguishable *in vivo* (Figures 1A,B,F, 2P).

### Morphogenesis During Binary Fission

#### Stomatogenesis and Development of Frontal-Ventral-Transverse Cirri During Cell Division

**FIGURE 3:**
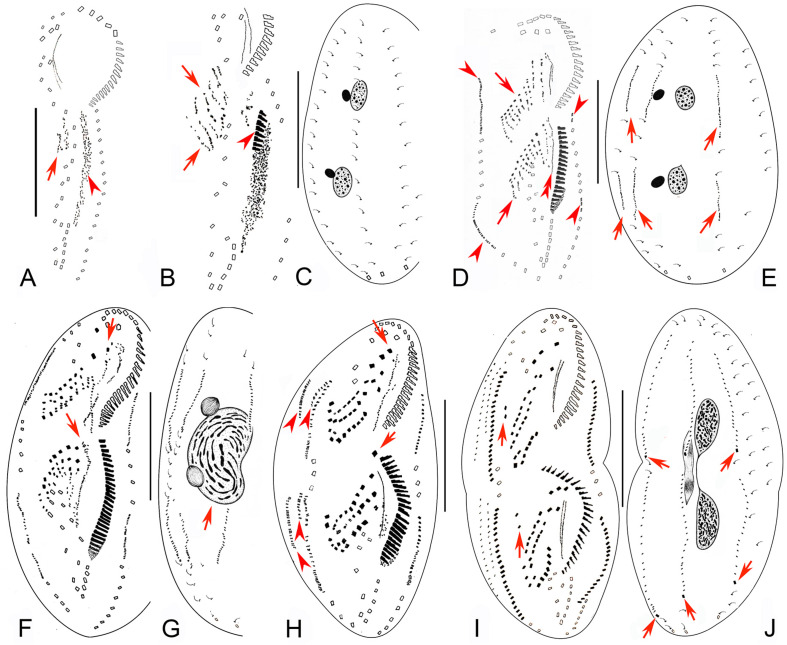
Early to middle-late morphogenetic stages of *Bourlandella viridis* (Kahl, 1932) comb. nov. **(A)** Ventral view of an early divider, to show the appearance of the oral primordium (arrowhead), arrow marks the frontal-ventral-transverse cirral anlagen (FVTA). **(B,C)** Ventral **(B)** and dorsal **(C)** views of the same divider, to show the streak-like FVTA (arrows), newly formed adoral membranelles (arrowhead), and the unchanged dorsal infraciliature **(C)**. **(D,E)** Ventral **(D)** and dorsal **(E)** views of the same early-middle divider, arrowheads in **(D)** show the marginal anlagen, arrows in **(D)** mark two sets of FVTA, double-arrowhead in **(D)** indicates the newly formed undulating membrane anlage in the opisthe, arrows in **(E)** indicate the dorsal kinety anlagen. **(F,G)** Ventral **(F)** and dorsal **(G)** views of a middle divider, showing the leftmost frontal cirri separated from anterior ends of undulating membranes anlagen (arrows in **F**) and the fused macronucleus (arrow in **G**). **(H)** Ventral view of a middle-late divider, showing the newly formed FVT cirri, arrows show the new leftmost frontal cirri, arrowheads indicate dorsomarginal anlagen. **(I,J)** Ventral **(I)** and dorsal **(J)** views of the same divider, showing the migration of the newly formed cirri, arrows in (I) indicate the frontoterminal cirri, arrows in **(J)** show the newly formed caudal cirri. Bars: 50 μm.

**FIGURE 4:**
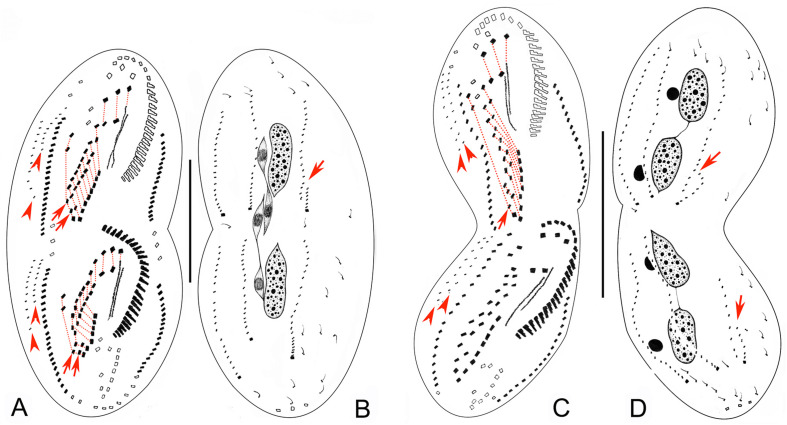
Late morphogenetic stages of *Bourlandella viridis* (Kahl, 1932) comb. nov. **(A,B)** Ventral **(A)** and dorsal **(B)** views of a later divider, to show the dorsomarginal anlagen (arrowheads) and the dorsal kinety fragmentation in proter (arrow in **B**), arrows in **(A)** indicate two pretransverse ventral cirri in both proter and opisthe, hatched lines show cirri originating from the same cirral anlage. **(C,D)** Ventral **(C)** and dorsal **(D)** views of a very late divider, to show the dorsomarginal kineties (arrowheads) and the dorsal kinety fragmentation (arrows in **D**), arrows in **(C)** indicate one pretransverse ventral cirrus in proter, hatched lines show cirri originating from the same cirral anlage. Bars: 50 μm.

**FIGURE 5:**
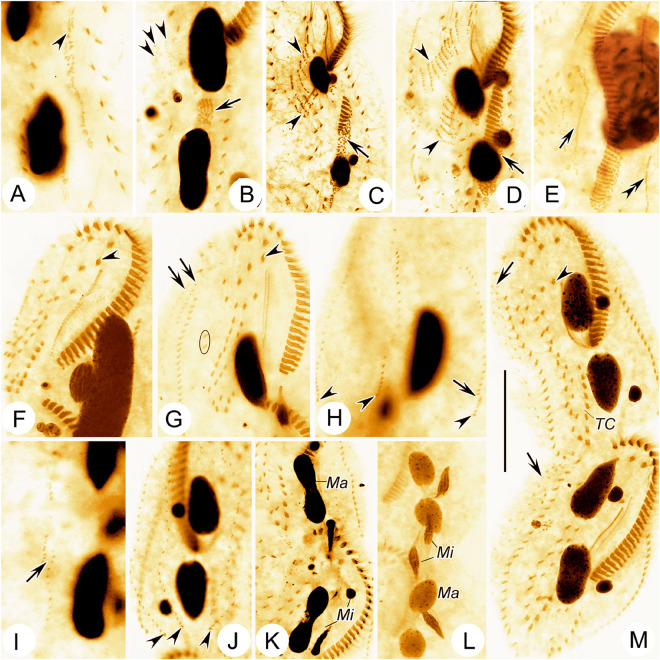
Photomicrographs of *Bourlandella viridis* (Kahl, 1932) comb. nov. during morphogenetic process (after protargol staining). **(A)** Ventral view of a very early divider, to show the oral primordium (arrowhead) in the opisthe. **(B)** Ventral view of an early divider, showing the new frontal-ventral-transverse cirral anlagen (FVTA, arrowheads) and the newly formed adoral membranelles (arrow). **(C)** Ventral view of another early divider, to show the streak-like FVTA (arrowheads) and newly formed adoral membranelles (arrow). **(D)** Ventral view of an early-middle divider, to show two sets of FVTA (arrowheads) and newly formed adoral membranelles (arrow). **(E)** Ventral view of a middle divider, showing the undulating membrane anlage (arrow) and left marginal anlage (double-arrowhead) in the opisthe. **(F)** Ventral view, to show the newly formed leftmost frontal cirrus (arrowhead). **(G)** Ventral view, to show the dorsomarginal anlagen (arrows), leftmost frontal cirrus (arrowhead), and frontoterminal cirri (in circle). **(H–J)** Dorsal views of different late dividers, to show newly formed caudal cirri (arrowheads) and the fragmentation of dorsal kinety 3 (arrow). **(K,L)** Different late dividers, showing the changes in the macronuclear nodules and micronuclei. **(M)** Ventral view of a very late divider, to show the newly formed cirri almost in their final positions, arrows show dorsomarginal kineties, arrowhead indicates buccal cirrus. Ma, macronuclear nodules; Mi, micronuclei; TC, transverse cirri. Bar: 50 μm.

In very early dividers, the oral primordium of the opisthe originates de novo alongside the left cirri of the parental midventral pairs between the adoral zone and the transverse cirral row (Figures 3A, 5A). Simultaneously, some right cirri of the parental midventral pairs below the level of the buccal cavity dedifferentiate to form the frontal-ventral-transverse cirral anlagen (FVTA) (Figures 3A, 5B). Subsequently, the oral primordium becomes larger by further proliferation of basal bodies and several membranelles form in the anterior portion (Figures 3B, 5B,C). During this process, some old midventral cirri dedifferentiate and become incorporated into the streak-like FVTA (Figures 3B, 5C). In the early-middle stage, the oral primordium of the opisthe continues to form new membranelles posteriad (Figure 5D) and the undulating membrane anlage (UMA or FVTA I) appears to the right of the oral primordium (Figures 3D, 5E). At the same time, the parental endoral begins to disintegrate and forms the UMA for the proter. The primary FVTA separate into two sets and begin to differentiate into new cirri (Figures 3F, 5E). From the middle-to-late stage, the formation of the oral primordium of the opisthe is gradually completed and the anterior part of the new adoral zone curves rightward; the parental adoral zone is completely retained by the proter. The UMA splits longitudinally into undulating membranes and also gives rise to the leftmost frontal cirrus in both the proter and the opisthe (Figures 3F,H, 5E–G). Simultaneously, other cirral anlagen break apart and differentiate into new cirri in the following pattern: FVTA II forms the middle frontal cirrus and the buccal cirrus; FVTA III forms the rightmost frontal cirrus and the parabuccal cirrus; FVTA IV to FVTA n–2 each forms one pair of midventral cirri and one transverse cirrus, although sometimes the anterior one to three anlagen do not form a transverse cirrus; FVTA n–1 forms one pair of midventral cirri, one pretransverse ventral cirrus and the penultimate transverse cirrus; FVTA n forms two frontoterminal cirri, one or two pretransverse ventral cirri, and the rightmost transverse cirrus (Figures 4A,C, 5M).

#### Formation of the Marginal Cirri, Dorsal Kineties, and Caudal Cirri During Cell Division

The right marginal anlagen develop intrakinetally as basal bodies derived from the disaggregated old marginal cirri and appear earlier than the left ones; formation of the left marginal anlagen commences in the same way as the right ones (Figures 3D,F, 5D,E). Then, all of the marginal anlagen lengthen toward both ends and generate new cirri that replace the old structures (Figures 3H,I, 4A,C, 5M).

New DK are formed in the typical *Oxytricha* pattern as two groups of primordia. One group develops intrakinetally within the parental structures as three thread-like anlagen in both the proter and the opisthe (Figures 3E,G). In late dividers, the rightmost anlage fragments to form two anlagen in the posterior region and, together with the two left anlagen, give rise to DK 1–4 (Figures 4B,D, 5H–J). The second group, which gives rise to the formation of the two dorsomarginal kineties, develops de novo to the right of the right marginal anlage in both the proter and the opisthe (Figures 3H,I, 4A,C, 5G,M). During the morphogenetic process, one caudal cirrus is formed at the posterior end of each of DK1, 2, and 4 (Figures 3J, 4B,D, 5H,J).

#### Division of Nuclear Apparatus During Cell Division

The process of nuclear division is typical for hypotrichs with two macronuclear nodules. The macronuclear nodules fuse into a single mass that splits into two elongated structures, one each in the proter and opisthe. Later, two macronuclear nodules are formed in each daughter cell (Figures 3C,E,G,J, 4B,D, 5K–M). The micronuclei divide mitotically.

### SSU rRNA Gene Sequence Characteristics and Phylogenetic Analyses

**FIGURE 6:**
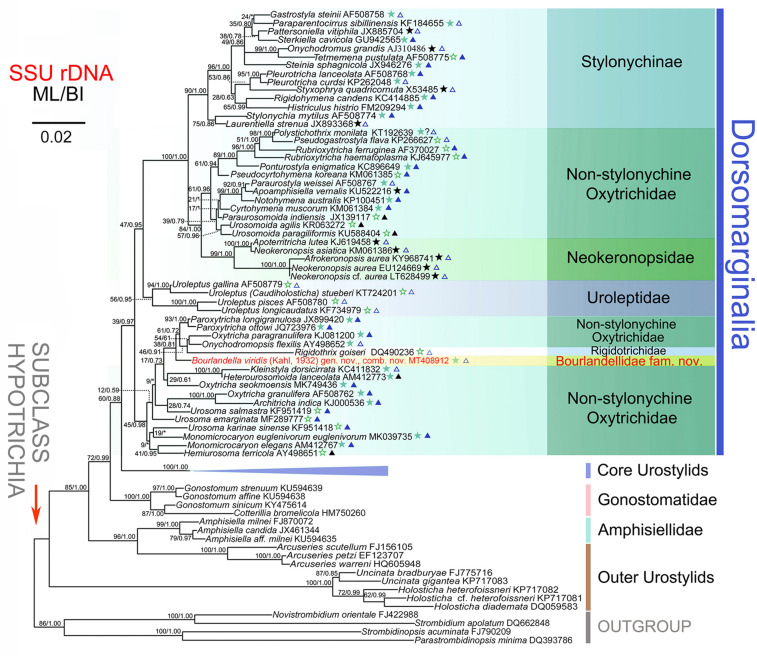
Maximum likelihood (ML) tree inferred from the SSU rRNA gene sequences, showing the systematic position of *Bourlandella viridis* (Kahl, 1932) comb. nov. (Indicated in red). Numbers at nodes denote the bootstrap values of maximum likelihood analysis (ML) and the posterior probabilities of Bayesian inference (BI) analysis. Asterisks indicate the disagreement between BI tree and ML tree. Stars mark dorsomarginalians with different fragmentation patterns (green solid stars, simple fragmentation; green hollow stars, without fragmentation; black solid stars, multiple fragmentation). Triangles indicate the number of frontal-ventral-transverse cirri of the dorsomarginalians (blue solid triangles, =18; blue hollow triangles, >18; black solid triangles, <18). All branches are drawn to scale. Scale bar corresponds to two substitutions per 100 nucleotide positions. GenBank accession numbers are given for each species.

The partial SSU rRNA gene sequence of *B. viridis* is deposited in GenBank with length, G+C content and accession number as follows: 1,680 bp, 45.8%, and MT408912. The topologies of the ML and BI trees are similar therefore only the ML tree is shown (Figure 6). *Bourlandella viridis* is nested within the dorsomarginalian assemblage and is sister to the *Paroxytricha longigranulosa* + *Paroxytricha ottowi* + *Oxytricha paragranulifera* + *Onychodromopsis flexilis* + *Rigidothrix goiseri* clade with low support values (ML/BI, 38/0.81), revealing that the position of *B. viridis* among the hypotrich ciliates is not robust. The SSU rRNA gene sequence similarities between *B. viridis* and members of its sister group ranges from 0.909 to 0.989.

## Discussion

### Identification of the Chinese Population of *Bourlandella viridis* and Comparision With Related Taxa

*Bourlandella viridis* was first reported as *Holosticha viridis* based on a population discovered near Hamburg, Germany by Kahl (1932). In the most recent work on the classification of *Holosticha*-like species, Li et al. (2017) established the genus *Limnoholosticha* and transferred *H. viridis* as well as three other urostylid species to this genus, although none of these four species had been described in detail. Consequently, it was noted that the genus diagnosis of *Limnoholosticha* is insufficient and needs to be improved pending the availability of further data (Li et al., 2017).

Here we provide a detailed description of a population that agrees very well with that of both the type population and another population of *B. viridis*, including the ovoidal to broad elliptical body shape, contractile vacuole in the mid-region of the body near the left margin, two macronuclear nodules each closely associated with a large micronucleus, lack of cortical granules, and the presence of endosymbiotic zoochlorellae (Kahl, 1932; Shen, 1983). Compared with the original population, our population has a slightly larger body size *in vivo* (100–130 μm vs. 100–110 μm), although this is within the range of variation that commonly occurs in populations from widely separated geographic regions (Berger, 1999, 2006). In the original description, Kahl (1932) mentioned the presence of five or six frontal cirri, however, he very likely mistook the buccal cirrus and frontoterminal cirri as frontal cirri. One characteristic that cannot be ignored is the variation in length of the dorsal bristles in the Qingdao population, the posterior ones being more than twice the length of the anterior ones. It is noteworthy that these bristles are distinct in differential interference contrast microscopy but inconspicuous in bright field microscopy. However, Kahl (1932) neither mentioned nor illustrated the dorsal bristles, probably because they are inconspicuous in bright field microscopy, and so he overlooked the longer bristles. Thus, we consider these two populations to be conspecific.

The presence of DK in an *Oxytricha* pattern suggests that *B. viridis* does not belong to the urostylids, but rather has a close relationship with the Dorsomarginalia. Moreover, *B. viridis* differs significantly from the type species of *Limnoholosticha*, *L*. *navicularum* (Kahl, 1932) Li et al., 2017, by having a smaller body size (100–130 vs. 200 μm), a different body shape (ovoidal to broad elliptical vs. elongate elliptical), two micronuclei each closely associated with a macronuclear nodule (vs. single micronucleus which lies between the two macronuclear nodules) and transverse cirri located 5/6 down the length of the cell (vs. transverse cirri located about two-thirds down the length of the cell) (Kahl, 1932; Berger, 2006). These features are widely used as genus-level characters in the taxonomy of hypotrichs, suggesting that *B. viridis* and *L*. *navicularum* should belong to different genera even though the dorsal kinety pattern of *L*. *navicularum* is not known (Berger, 1999, 2006; Foissner, 2016). *Bourlandella viridis* can be separated from *L*. *algivora* (Kahl, 1932) Li et al., 2017 in the following aspects: (1) body size *in vivo* larger (100–130 × 50–60 μm from raw cultures vs. 74 μm in length); (2) endosymbiotic algae present and distributed evenly throughout the cell (vs. absent, cell color due to ingested green algae, especially euglenids); and (3) cortical granules absent (vs. rows of colorless cortical granules present) (Kahl, 1932). *Bourlandella viridis* can be easily distinguished from *L*. *setifera* (Kahl, 1932) Li et al., 2017 by: (1) body shape (ovoidal to broad elliptical vs. slender elongate-elliptical); (2) each macronuclear nodule is associated with a micronucleus (vs. two macronuclear nodules with single micronucleus in between); (3) number of transverse cirri (on average eight vs. five); (4) endosymbiotic algae present (vs. absent); and (5) freshwater (vs. brackish water) habitat (Kahl, 1932).

**FIGURE 7:**
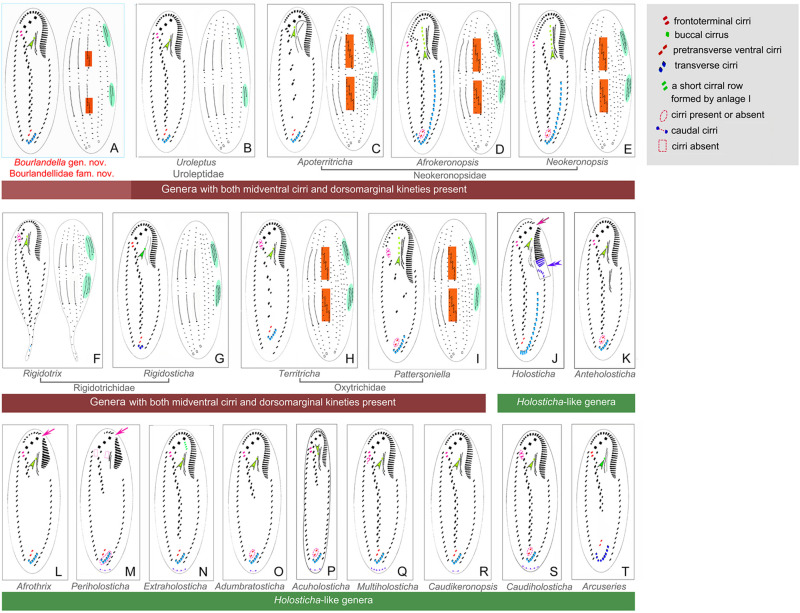
Infraciliature patterns of the genera morphologically similar to *Bourlandella* gen. nov. (**B–I**, ventral and dorsal views of the midventral dorsomarginal genera; **J–T**, ventral views of the *Holosticha*-like genera). **(A)** *Bourlandella* gen. nov. **(B)** *Uroleptus*. **(C)** *Apoterritricha*. **(D)** *Afrokeronopsis*. **(E)** *Neokeronopsis*. **(F)** *Rigidothrix*. **(G)** *Rigidosticha*. **(H)** *Territricha*. **(I)** *Pattersoniella*. **(J)** *Holosticha*. **(K)** *Anteholosticha*. **(L)** *Afrothrix*. **(M)** *Periholosticha*. **(N)** *Extraholosticha*. **(O)** *Adumbratosticha*. **(P)** *Acuholosticha*. **(Q)** *Multiholosticha*. **(R)** *Caudikeronopsis*. **(S)** *Caudiholosticha*. **(T)** *Arcuseries*. Dorsal kinety fragmentation and dorsomarginal kineties are shaded in red and green, respectively. Arrows indicate the gap in the adoral zone of membranelles, arrowheads show the buccal cirrus, double-arrowhead indicates the special structures of anterior end of left marginal row curved rightward and posterior adoral membranelles of proximal portion wider than remaining ones.

With respect to the cirral pattern (three frontal cirri, single marginal row on each body side and midventral complex comprized of midventral pairs only), 11 *Holosticha*-like genera should be compared with *Bourlandella* gen. nov. (Berger, 2006; Li et al., 2017), although all of these can be easily separated from *Bourlandella* gen. nov. by key morphological characters as shown in Figures 7J–T. Among these genera, *Multiholosticha* Li et al., 2017 has six DK like *Bourlandella* gen. nov., However, there is a single caudal cirrus at end of each kinety in *Multiholosticha multicaudicirrus* (Song and Wilbert, 1989) Li et al., 2017, and a curved row of 14–16 caudal cirri present in *Multiholosticha interrupta* (Dragesco, 1966) Li et al., 2017, indicating that dorsomarginal kineties and dorsal kinety fragments are probably absent in *Multiholosticha* (vs. present, in *Bourlandella* gen. nov.) (Dragesco, 1966; Song and Wilbert, 1989; Berger, 2006).

Eight genera (*Afrokeronopsis* Foissner and Stoeck, 2008, *Apoterritricha* Kim et al., 2014, *Neokeronopsis* Warren et al., 2002, *Pattersoniella* Foissner, 1987, *Rigidosticha*, *Rigidothrix*, *Territricha* Berger and Foissner, 1988, and *Uroleptus*) resemble *Bourlandella* gen. nov. in possessing both a midventral complex with a zigzag row of cirral pairs and dorsomarginal kineties, although these genera can be separated from the latter by various morphological characters as shown in Figures 7B–I (Berger, 1999; Warren et al., 2002; Foissner and Stoeck, 2006, 2008; Foissner et al., 2010; He et al., 2011; Huang et al., 2014; Kim et al., 2014; Bharti et al., 2016; Chen et al., 2016).

### Redefinition of the Family Rigidotrichidae Foissner and Stoeck, 2006 and Establishment of Bourlandellidae Nov. Fam.

Among the dorsomarginalian hypotrichs with a midventral complex, eight genera (*Afrokeronopsis*, *Apoterritricha*, *Neokeronopsis*, *Pattersoniella*, *Rigidosticha*, *Rigidothrix*, *Territricha*, and *Uroleptus*) have characters that are typical of more than one family and show incongruence between their morphological and molecular data. Consequently, these genera have had a confused taxonomic and phylogenetic history. The family Rigidotrichidae is characterized by having an oxytrichid frontal ciliature, dorsomarginal kineties, and a midventral complex with cirral pairs arranged in a zigzag pattern. It was erected for the curious species *R. goiseri* Foissner and Stoeck, 2006, which possesses a rigid body and a distinct midventral cirral pattern (Foissner and Stoeck, 2006). Three other genera, i.e., *Uroleptus*, *Territricha*, and *Afrophrya* Foissner and Stoeck, 2006, were also initially assigned to the family Rigidotrichidae by Foissner and Stoeck (2006). Based on the CEUU hypothesis and molecular analyses, Foissner and Stoeck (2008) transferred *Uroleptus* from Rigidotrichidae to the newly established family Uroleptidae Foissner and Stoeck, 2008, which was diagnosed as a group of very flexible midventral hypotrichs that form a distinct clade within the oxytrichids in molecular phylogenetic trees. Bharti et al. (2016) described a new rigidotrichid genus, *Rigidosticha*. Consequently, the family Rigidotrichidae currently includes four genera, namely *Rigidothrix*, *Territricha*, *Afrophrya*, and *Rigidosticha*., However, *Rigidothrix* and *Rigidosticha* differ from *Territricha* by the following combination of family-level characters: (1) body rigid (vs. flexible), (2) dorsal kinety fragmentation absent (vs. present); (3) distinct (vs. indistinct) zigzag midventral pattern; and (4) cortical granules/extrusomes absent (vs. present) (Berger, 1999; Foissner and Stoeck, 2006; Bharti et al., 2016). Based on the above differences, we suggest that *Territricha* should be excluded from Rigidotrichidae for which an improved diagnosis is provided. As there is no information about dorsomarginal kineties or dorsal kinety fragmentation for *Afrophrya*, we tentatively accept the classification of *Afrophrya* suggested by Foissner and Stoeck (2006) until more information is available, in particular concerning its dorsal ciliature and molecular phylogeny.

#### Improved Diagnosis for Rigidotrichidae Foissner and Stoeck, 2006

Rigid or flexible Dorsomarginalia with three frontal cirri; midventral cirri arranged in zigzag pattern; frontoterminal cirri present; dorsal kinety fragmentation absent; ontogenesis in urostylid-mode with FVT-cirri formed in more than six anlagen.

#### Type Genus

*Rigidothrix* Foissner and Stoeck, 2006

#### Genera Assignable

*Rigidothrix* Foissner and Stoeck, 2006; *Afrophrya* Foissner and Stoeck, 2006; *Rigidosticha* Bharti et al., 2016

Hitherto, three families have been established for species with both midventral cirri and dorsomarginal kineties, namely Rigidotrichidae, Uroleptidae, and Neokeronopsidae Foissner and Stoeck, 2008. As *B. viridis* possesses simple kinety fragmentation, it should not be assigned to either Rigidotrichidae or Uroleptidae, members of which lack kinety fragmentation, and neither should it be assigned to Neokeronopsidae, which is characterized by multiple kinety fragmentation. In the SSU rRNA gene tree, *B. viridis* is sister group to the *P. longigranulosa* + *P. ottowi* + *O. paragranulifera* + *O. flexilis* + *Rigidothrix* clade and is placed far from the Uroleptidae and Neokeronopsidae clades. Consequently, based on both morphological and molecular analyses, we suggest that *B. viridis* represents a separate family and a new family, Bourlandellidae fam. nov., is established (for definition, see “Results” section).

### Ontogenetic Comparision

The morphogenetic process of *B. viridis* is here compared with that of genera with both midventral cirri arranged in a zigzag pattern and dorsomarginal kineties present (referred to as “midventral dorsomarginalian genera” in the following text). To date, ontogenetic processes have been reported for six midventral dorsomarginalian genera, i.e., *Afrokeronopsis*, *Apoterritricha*, *Neokeronopsis*, *Pattersoniella*, *Rigidothrix*, and *Uroleptus* (Berger, 1999; Warren et al., 2002; Foissner and Stoeck, 2006, 2008; Foissner et al., 2010; He et al., 2011; Kim et al., 2014; Chen et al., 2016). Their available morphogenetic features show high similarities with *B. viridis*, that is: (1) the parental adoral zone of membranelles (AZM) is completely inherited by the proter; (2) some parental midventral cirri participate in the formation of the FVTA in the urostylid-mode, i.e., in more than six anlagen; (3) the FVTA are formed in a primary pattern, that is one group of anlagen is formed at the beginning of the process and this subsequently splits into two sets of anlagen, one for the proter, the other for the opisthe; (4) new marginal anlagen develop within each parental row; (5) the new DK originate from two groups of primordia, i.e., one group develops intrakinetally within the old structures, whereas the other one originates de novo at the anterior end of the right marginal anlagen to form the dorsomarginal kineties; and (6) all macronuclear nodules fuse into single mass which then divides.

The most significant ontogenetic feature shared by the midventral dorsomarginalian genera is that the FVT anlagen are generated in the urostylid-mode, that is more than six anlagen are formed from which the midventral cirri are developed. The zigzag midventral cirral pattern, which used to be considered as a diagnostic feature for the urostylids (a non-dorsomarginalian group), is now also known to be widespread among dorsomarginalian taxa (Berger, 2006; Foissner and Stoeck, 2006, 2008; Foissner et al., 2010; Kim et al., 2014; Chen et al., 2016). The oxytrichids, which constitute the main group within the Dorsomarginalia, usually have 18 FVT cirri clustered in groups and which originate from six FVT anlagen (Berger, 1999, 2008).

Another noteworthy feature of the midventral dorsomarginalian genera is that the parental AZM is inherited completely intact by the proter. Interestingly, this feature is also shared by all the other dorsomarginalian members. In contrast, the parental AZM of the urostylids has various fates during morphogenesis: (1) for most of the “core urostylids” and the *Arcuseries* species (members of the “outer urostylids”), the parental AZM is replaced by the newly built oral primordium; (2) for the other “core urostylids”, the proximal portion of the parental AZM shows distinct signs of reorganization; and (3) for the *Holosticha* species, which are members of the “outer urostylids,” the parental AZM is retained by the proter (Berger, 2006; Song and Shao, 2017; Chen et al., 2020).

Although all midventral dorsomarginalian genera have dorsomarginal kineties, the presence or absence of dorsal kinety fragmentation and the pattern of this fragmentation varies among them. Both *Rigidothrix* and *Uroleptus* lack dorsal kinety fragmentation during the ontogenetic process. *Afrokeronopsis*, *Apoterritricha*, *Neokeronopsis*, and *Pattersoniella* show multiple fragmentation of DK3 (the rightmost dorsal kinety), whereas simple fragmentation of DK3 occurs in *Bourlandella* gen. nov. Among the dorsal kinety fragmentation patterns in dorsomarginalians, simple fragmentation of DK3 is the most common. Other patterns might have developed by modification of simple DK3 fragmentation. For example, multiple fragmentation might have developed thereby increasing the number of fragments; the *Tachysoma* pattern might have developed by adding fragmentation of another dorsal kinety; and the absence of fragmentation might be due to its loss. None of the “core urostylids” shows kinety fragmentation. In contrast, dorsal kinety fragmentation occurs in *Holosticha heterofoissneri*, a member of the “outer urostylids”, in a pattern that differs from those of the dorsomarginalians, i.e., the leftmost kinety (DK1) fragments (vs. the rightmost one in dorsomarginalians) (Berger, 2006; Song and Shao, 2017).

### Phylogenetic Perspectives

As shown in the present phylogenetic tree (Figure 6), *B. viridis* is sister to the *P. longigranulosa* + *P. ottowi* + *O. paragranulifera* + *O. flexilis* + *R. goiseri* clade. Among the species of this clade, *R. goiseri* most closely resembles *B. viridis*, that is, both are dorsomarginalian taxa with midventral pairs. However, *R. goiseri* differs from *B. viridis* by having a rigid (vs. flexible) body and the absence (vs. presence) of dorsal kinety fragmentation (Bharti et al., 2016). The two *Paroxytricha s*pecies and *O. paragranulifera* can be easily distinguished from *B. viridis* by their typical 18 FVT cirral pattern (vs. zigzag midventral cirral pattern) (Berger, 1999; Shao et al., 2014; Foissner, 2016). Unlike *B. viridis*, *O. flexilis* has several marginal rows (vs. one right and one left marginal row) and a modified oxytrichid cirral pattern, that is, frontoventral cirri in a short transverse row or lacking and postoral ventral cirri in a longitudinal row right of the buccal vertex (vs. in typical zigzag midventral cirral pattern) (Berger, 1999).

Traditionally the taxonomy of hypotrichs was mainly based on the patterns of distribution of the ventral cirri, e.g., zigzag midventral cirral pattern of urostylids, 18 frontal-ventral-transverse cirri of oxytrichids (Kahl, 1932; Borror, 1972; Corliss, 1977, 1979; Jankowski, 1979; Lynn and Small, 2002). In recent years, however, it has become apparent that the dorsal infraciliature is often as informative as the ventral cirral pattern, particularly for higher-rank classification (Berger, 1999, 2006; Foissner et al., 2004; Li et al., 2017). One example is the presence or absence of dorsomarginal kineties, based partly on which Berger (2006) divided the Hypotrichia into Urostyloidea (midventral cirri arranged in a zigzag pattern, dorsomarginal kineties absent) and Dorsomarginalia (dorsomarginal kineties present). These features are, however, sometimes shared by species with intermediate morphologies, e.g., dorsomarginal taxa with midventral cirri arranged in a zigzag pattern (*Afrokeronopsis*, *Apoterritricha*, *Neokeronopsis*, *Pattersoniella*, *Rigidosticha*, *Rigidothrix*, *Territricha*, and *Uroleptus*). Based on their cirral pattern these genera should be classified as urostylids, but molecular phylogenies have revealed close evolutionary relationships with the oxytrichids. Under this framework, it is postulated that these genera retained their dorsomarginal kineties, which is a conserved feature, whereas their ventral and remaining dorsal infraciliature continued to evolve. On the ventral side, six or more basic anlagen generate the FVT cirral patterns, among which the conspicuous 18 FVT cirral pattern was thought to be the dominant pattern of the oxytrichid dorsomarginal taxa (Berger, 1999, 2006; Song and Shao, 2017). Hence, the eight dorsomarginalian genera with midventral cirri arranged in a zigzag pattern become inexplicable exceptions. To resolve this dilemma, Foissner et al. (2004) proposed the CEUU hypothesis which posited that the zigzag midventral pattern probably evolved twice from the 18 FVT cirral pattern of an oxytrichine ancestor by anlagen multiplication.

Historically, oxytrichids were thought to have derived from ancestors of the typical urostylid genus, *Urostyla* (Kahl, 1932; Borror, 1972). The findings of the present study are consistent with this hypothesis. As shown in the SSU rRNA gene tree (Figure 6), some species with midventral cirri arranged in a zigzag pattern (e.g., *Holosticha* spp. and *Uncinata* spp.), treated as “outer urostylids”, diverged basally within the subclass Hypotrichia, followed by the *Arcuseries* (“outer urostylids”) + *Amphisiella* clade, the gonostomatids, the “core urostylids” and finally the Dorsomarginalia. Following the clade represented by the majority of uroleptids, the first clade that diverges in the Dorsomarginalia comprises *B. viridis* and various other species, albeit with unstable support (ML/BI, 45/0.98). A fully supported and stable clade comprising various sub-clades branches last. Within this latter clade, the monophyletic Stylonychinae is sister group to the sub-clade comprising the monophyletic Neokeronopsidae and several non-stylonychine oxytrichids. From the SSU rRNA gene tree (Figure 6) it can also be inferred that oxytrichids with a grouped FVT cirral pattern (i.e., the main group of dorsomarginalians) might have evolved from a urostylid ancestor by decreasing the number of FVT anlagen several times. Therefore, so-called intermediate species, i.e., dorsomarginalians with midventral cirri arranged in a zigzag pattern such as the uroleptids, neokeronopsids, *R. goiseri*, and *B. viridis*, probably inherited their midventral cirral pattern from the urostylids.

The dorsal kinety fragmentation patterns (see above) differ among the dorsomarginalian species. Previous studies suggested that the dorsal kinety fragmentation patterns carry a phylogenetic signal, e.g., the Oxytrichidae was mainly defined by the oxytrichid dorsal kinety fragmentation (simple fragmentation of DK3) (Berger, 1999, 2006, 2008). However, species with the same fragmentation patterns did not always cluster together within the SSU rRNA gene tree (Figure 6), revealing that the fidelity of this phylogenetic signal is unstable in our analyses.

### Contribution of Mixotrophy

Mixotrophy is widespread among freshwater ciliates as a result of the autotrophic activities either of endosymbiotic zoochlorellae or sequestered chloroplasts from ingested algae (Berninger et al., 1986; Foissner et al., 1999; Chao and Katz, 2008). The best known zoochlorellae–bearing ciliates are *Paramecium bursaria*, *Ophrydium versatile*, and *Euplotes daidaleos*, and remarkable instances of chloroplast sequestration are represented by *Histiobalantium natans* and *Strombidium viride* (Esteban et al., 2010). The evolution of mixotrophy in ciliates has been a complex mixture of events, each representing a major evolutionary transition (Dolan, 1992). For instance, the presence of zoochlorellae is a distinctive character for the identification of *P. bursaria*. Furthermore, the presence of intracellular photoautotrophs could help the host ciliates to increase their growth rates, reduce their mortality and adapt to nutrient-poor environments (Dolan, 1992; Esteban et al., 2010). Thus, the presence or absence of mixotrophy is an informative signal for ciliate systematics.

The species-group name “viridis” refers to the presence of zoochlorellae within the host ciliate (Kahl, 1932). Freshly isolated cells of *B. viridis* invariably possess conspicuous endosymbiotic zoochlorellae (Figures 1A,C, 2C,D), and the ciliates can survive for at least half a month in culture without additional bacterial food so it appears that this species is mixotrophic and the algae provide some organic matter to it. However, mixotrophy in *B. viridis* might be temporary since, when kept in laboratory culture for prolonged periods of time (more than 1 month), the ciliate digests almost all of its endosymbionts and appears colorless (Figures 2A,B,E). The same phenomenon occurs in *P. bursaria* when maintained in the laboratory in the absence of light and food (Dolan, 1992). Currently we do not know the mixotrophic strategy adopted by *B. viridis*, i.e., whether it has a stable relationship with its endosymbiotic zoochlorellae or if it sequesters and enslaves algal chloroplasts. Further studies are therefore needed in order to address questions such as: (1) What is the mixotrophic strategy of *B. viridis*? (2) How important is photoautotrophy for nutrient acquisition in *B. viridis*? (3) Does mixotrophy allow *B. viridis* to adapt to new environments?

## Data Availability Statement

The datasets presented in this study can be found in online repositories. The names of the repository/repositories and accession number(s) can be found from the GenBank database: MT408912.

## Author Contributions

XTL and YZ conceived and designed the manuscript. WYS and TYZ carried out the live observation, protargol staining, and analyzed the data. WYS, TYZ, XZ, AW, WBS, XTL, and YZ wrote the manuscript. All authors contributed to the article and approved the submitted version.

## Conflict of Interest

The authors declare that the research was conducted in the absence of any commercial or financial relationships that could be construed as a potential conflict of interest.

## Footnotes

1 http://guidance.tau.ac.il/

2 http://www.phylo.org
