# Supplementary material for: Taxonomy, Ontogenesis and Evolutionary Relationships of the Algae-Bearing Ciliate Bourlandella viridis (Kahl, 1932) comb. nov., With Establishment of a New Genus and New Family (Protista, Ciliophora, Hypotrichia)
Source: Front Microbiol. 2021 Jan 28;11:560915. doi: 10.3389/fmicb.2020.560915 (PMC7875870; doi:10.3389/fmicb.2020.560915)
Supplement: Supplementary file 1 [file Table_1.DOCX]

**Supplementary table 1**. Genbank accession numbers of core urostylids included in phylogenetic analyses that are not shown in Figure 6

| Species | GB number | Species | GB number |
| --- | --- | --- | --- |
| *Anteholosticha* cf. *azerbaijanica* | FJ775712 | *Bakuella subtropica* | KY874001 |
| *Anteholosticha manca* | DQ503578 | *Bergeriella ovata* | FJ754026 |
| *Anteholosticha marimonilata* | FJ870075 | *Diaxonella trimarginata* | JQ424833 |
| *Anteholosticha monilata* | GU942567 | *Extraholosticha sylvatica* | KJ958490 |
| *Anteholosticha multicirrata* | KC307773 | *Hemicycliostyla franzi* | AM412765 |
| *Anteholosticha paramanca* | KF806443 | *Hemicycliostyla sphagni* | FJ361758 |
| *Anteholosticha pseudomonilata* | HM568416 | *Heterokeronopsis pulchra* | JQ083600 |
| *Anteholosticha pulchra* | KF306393 | *Holosticha polystylata* | AF508760 |
| *Anteholosticha randani* | KU234524 | *Metaurostylopsis struederkypkeae* | JQ424832 |
| *Anteholosticha rectangula* | KU175624 | *Neobakuella flava* | GU967698 |
| *Anteholosticha songi* | MK713372 | *Nothoholosticha fasciola* | FJ377548 |
| *Antiokeronopsis flava* | KF806444 | *Pseudokeronopsis erythrina* | FJ775723 |
| *Apobakuella fusca* | JN008942 | *Pseudourostyla subtropica* | KR013239 |
| *Apoholosticha sinica* | KJ000285 | *Thigmokeronopsis stoecki* | EU220226 |
| *Apokeronopsis ovalis* | EU930048 | *Trichototaxis marina* | KJ845346 |
| *Apokeronopsis sinica* | FJ461474 | *Uroleptopsis citrina* | GU437211 |
| *Australothrix xianiensis* | KT892731 | *Urostyla grandis* | AF508781 |
